# Photochemical Isomerization and Topochemical Polymerization of the Programmed Asymmetric Amphiphiles

**DOI:** 10.1038/srep28659

**Published:** 2016-06-24

**Authors:** Dae-Yoon Kim, Sang-A Lee, Daseal Jung, Kwang-Un Jeong

**Affiliations:** 1 BK21 Plus Haptic Polymer Composite Research Team, Polymer Materials Fusion Research Center, and Department of Polymer-Nano Science and Technology, Chonbuk National University, Jeonju, Jeonbuk 54896, Korea

## Abstract

For the advancement in multi-stimuli responsive optical devices, we report the elaborate molecular engineering of the dual photo-functionalized amphiphile (abbreviated as AZ_1_DA) containing both a photo-isomerizable azobenzene and a photo-polymerizable diacetylene. To achieve the efficient photochemical reactions in thin solid films, the self-assembly of AZ_1_DA molecules into the ordered phases should be precisely controlled and efficiently utilized. First, the remote-controllable light shutter is successfully demonstrated based on the reversible *trans*-*cis* photo-isomerization of azobenzene group in the smectic A mesophase. Second, the self-organized monoclinic crystal phase allows us to validate the photo-polymerization of diacetylene moiety for the photo-patterned thin films and the thermo-responsible color switches. From the demonstrations of optically tunable thin films, it is realized that the construction of strong relationships between chemical structures, molecular packing structures and physical properties of the programmed molecules is the core research for the development of smart and multifunctional soft materials.

Since the physical properties of soft materials strongly depend on their molecular conformations and organizations, understanding the intermolecular physical bonds between the chemically connected building blocks is an essential prerequisite to manipulate the well-defined molecular architectures^1^,^2^. Amphiphilic supramolecules adopting the shape of lipids have been considered as the chemistry beyond molecules because their asymmetric characteristics allow us to form the dimensionally controllable nanostructures through the non-covalent interactions and the nanophase separations^3^,^4^. Self-organizations into the long-range ordered hierarchical superstructures, such as lamellar-, micellar-, and fibrillar-aggregates, exhibit attractive properties for nonlinear optics, interface stabilizers, and biochemical sensors^5^,^6^,^7^,^8^.

Recently, multitudinous functional groups can be cooperatively utilized into the amphiphilic supramolecules to achieve the targeted properties of intelligent materials, such as ionic liquid gels, polymeric giant surfactants, photoluminescence liquid crystals, and lyotropic photonic crystals^9^,^10^,^11^,^12^. Among them, the diacetylene-functionalized amphiphiles have increased interests because the topochemical polymerization can generate the conjugated polymer networks by simply irradiating the 254 nm UV light^13^. In addition, the color change from blue to red by tuning the effective conjugation length is another noticeable property of polydiacetylene, which can be utilized in the potential applications for photovoltaic cells, bioelectronic chips and colorimetric sensors^14^. With the azobenzene-functionalized amphiphiles, the formation and deformation of self-assembled hierarchical superstructures can be modulated by the photochemical isomerization upon irradiating the 365 nm UV and 450 nm Vis lights^15^. The individual azobenzene conformational changes are translated to the macroscopic amplifications resulting in the transformation of molecular packing symmetry^16^. The photo-reversible property of azobenzene has been the basis for smart materials with remote-controllable applications in switchable delivery systems, rewritable hologram films, and optically active fibers^17^,^18^,^19^.

The main objective of this study is to fully utilize the different photo-functional groups on a single molecule that can be beneficial for the fabrication of optically tunable multifunctional thin films. Since the light-driven phase structural behaviors strongly rely on their molecular packing structures, understanding the supramolecular assembly of photo-functionalized amphiphile is significantly important^20^. Note that diacetylene-based molecules in the highly ordered phases only undergo the topochemical polymerization^21^. Contradistinctively, in the case of azobenzene-based molecules, it is widely accepted that the photochemical isomerization in the highly ordered phase is basically suppressed and limited due to the lack of free volume but allowed in the partially ordered mesophases^22^. Therefore, the manipulation of hierarchical superstructures with the fine control of intermolecular interactions between the programmed molecular building blocks are very important to achieve the targeted properties of soft materials^23^,^24^,^25^.

Herein, we newly introduce a dual photo-functionalized amphiphile (abbreviated as AZ_1_DA) containing both azobenzene and diacetylene groups. The first strategy for the molecular design of AZ_1_DA is the self-organization of asymmetric supramolecule into the low-ordered liquid crystalline (LC) phase as well as into the highly ordered crystalline (Cr) phase. Introducing the photo-responsive groups into the AZ_1_DA is the second strategy for the molecular programming for the photochemical isomerization and the topochemical polymerization. After studying the ordered molecular arrangements of AZ_1_DA with the combined techniques of thermal, microscopic, scattering, and simulation, the photochemical isomerization and the topochemical polymerization of AZ_1_DA are investigated for the demonstration of remote-controllable light shutter, photo-patterned thin film, and thermo-responsive color switch.

## Results and Discussion

### Programmed amphiphile and its supramolecular assembly

**Figure 1:**
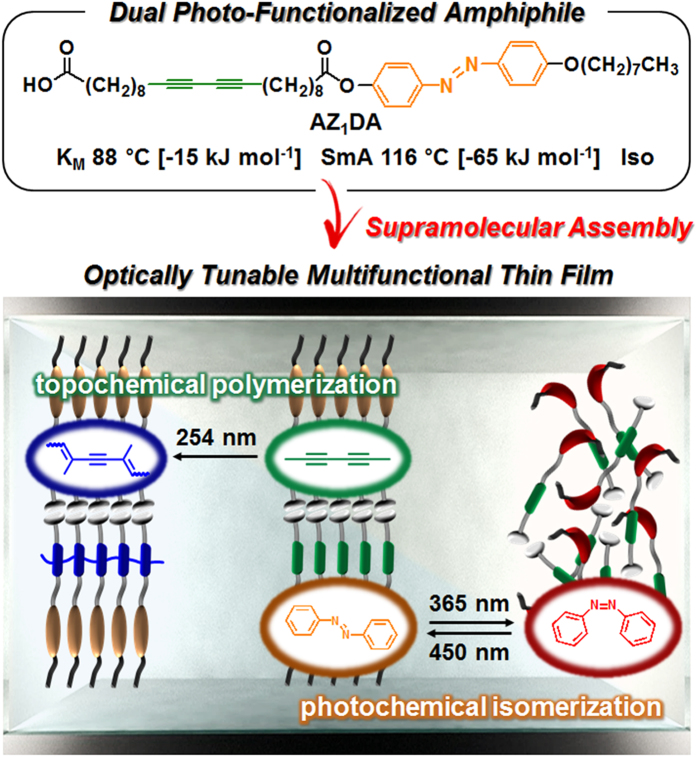
Programmed asymmetric amphiphile. Chemical structures of dual photo-functionalized amphiphile and self-assembly pathways of AZ_1_DA supramolecules in the ordered phases for fully utilizing the photochemical isomerization and topochemical polymerization in the thin films.

To develop the optically tunable multifunctional thin films, we report the programmed asymmetric amphiphile with photo-isomerizable azobenzene group and the photo-polymerizable diacetylene moiety (named AZ_1_DA, Fig. 1). The azobenzene is purposely chosen as the photo-responsive mesogen because it is useful to build the ordered structures and to control them by light. By introducing the diacetylene group, it is easy to prepare the conjugated polymers with characteristics of thermo-responsive behaviors, which cannot be synthesized by any alternative methods^26^. The carboxylic head group is a well-known building block for enhancing supramolecular aggregates due to the intermolecular hydrogen (*H*)-bondings. The hydrophobic alkyl chains chemically attached to the both sides of azobenzene and diacetylene tend to induce the nanophase separation. Therefore, we expect that the AZ_1_DA compound can be easily adopted to validate the optical property changes depending on the supramolecular packing structures by tuning the selective wavelengths of light for the photochemical isomerization and the topochemical polymerization. Detail synthetic procedure of AZ_1_DA and chemical structure and purity of the target compound is confirmed by NMR, MALDI-ToF and elemental analysis (see Supplementary Figs S1–S5).

Phase transition behaviors of the AZ_1_DA compound are first investigated by DSC and POM (see Supplementary Fig. S6). Two thermal transitions are detected at 116.5 °C and 88.7 °C during cooling. The thermal properties obtained from the subsequent heating process are consistent with those from the cooling process, which means the existence of enantiotropic LC mesophase. Upon heating the AZ_1_DA compound, the highly ordered Cr phase melts first at the lower temperature, and the partially ordered LC phase turns to the disordered Iso phase at the higher temperature. As cooling from the Iso phase, a typical fan-shaped texture of LC phase is observed at 100 °C. Further decreasing the temperature to 25 °C results in the strong birefringent aggregates, which is often observed during a crystallization process^27^. The overall transition temperatures with the corresponding heat of transitions are additionally summarized in Fig. 1.

**Figure 2:**
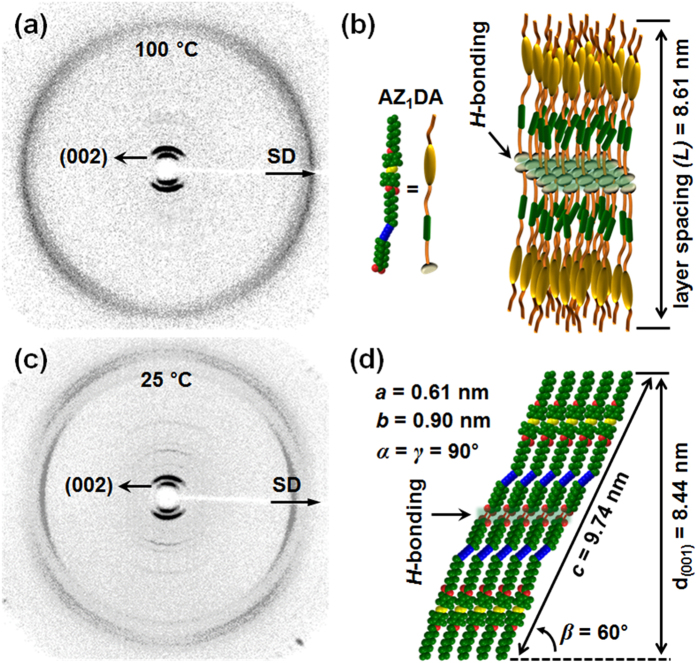
Molecular packing structure. 2D WAXD patterns of the oriented AZ_1_DA in the LC phase at 100 °C (**a**) and the Cr phase at 25 °C (**c**) and their corresponding schematic illustration of molecular packing structures of the LC (**b**) and Cr (**d**) phases, respectively.

To investigate the molecular packing symmetries in the ordered phases, structure sensitive SAXS and WAXD experiments are conducted at different temperatures^28^. As shown in Fig. 2(a), a pair of diffraction peak at 2θ = 2.05° on the meridian is observed by irradiating X-ray normal to the shear direction (SD) of the macroscopically oriented AZ_1_DA sample in the LC phase. Three sharp and intense peaks at 2θ = 1.02° (d = 8.61 nm), 2.05° (d = 4.30 nm) and 3.08° (d = 2.87 nm) are detected in the low-angle region which possess the *q*-value ratio of 1:2:3 (see Supplementary Fig. S7). This result supports the formation of partially ordered smectic (Sm) LC phase in which the AZ_1_DA molecules are stacked into layers with the liquid-like short-range positional order^29^. However, the higher value of layer spacing (*L* = 8.61 nm) than theoretical length (*l* = 4.89 nm) of the AZ_1_DA molecule indicates the fact that the layer structure is constructed from more than one molecule (see Supplementary Fig. S8). Note that the AZ_1_DA molecule can be dimerized *via* the intermolecular interaction of carboxylic polar groups^30^. The head-to-head dimer formation of AZ_1_DA molecules through the *H*-bonding in the LC phase can be supported by the observation of C = O stretching vibration between 1685 and 1705 cm^−1^ in the FT IR measurements (see Supplementary Fig. S9). Since the weak and diffused halo at 2θ = 20.9° (d = 0.42 nm) originated from the average periodicity of electron density between the nanophase separated azobenzene mesogens and diacetylene moieties is on the equator, the Sm layer should be interdigitated and the average long axis of the dimer is parallel to the layer normal direction. Based on the combined experimental results, this low-ordered LC structure is identified as a smectic A (SmA) phase, as illustrated in Fig. 2(b).

As shown in Fig. 2(c), a pair of diffraction peak at 2θ = 2.09° (d = 4.21 nm) is clearly detected with its higher order diffractions on the meridian in the Cr phase. Miller indices of the diffractions at 2θ = 1.04°, 2.09°, 3.12°, 7.28°, 8.33° and 11.4° are identified as (001), (002), (003), (007), (008) and (0011), respectively. Therefore, the layered structure is maintained even at 25 °C and the layer normal is perpendicular to the SD. On the equator, the diffraction at 2θ = 19.7° (d = 0.45 nm) indicates that a highly ordered 3D structure is formed by the lateral molecular close packings. From the diffractions at 2θ = 21.4° (d = 0.41 nm) and 2θ = 22.1° (d = 0.40 nm) in the quadrants, it is realized that the dimeric AZ_1_DA building blocks are tilted from the layer normal direction^31^. Careful structural analysis of the oriented AZ_1_DA gives a monoclinic unit cell with the dimensions of *a* = 0.61 nm, *b* = 0.90 nm, *c* = 9.74 nm, *α* = 90.0°, *β* = 60.0° and *γ* = 90.0° via the refinement of the reciprocal lattice, and this Cr phase is abbreviated to be the K_M_ phase. From the crystallographic point of view, the head-to-head AZ_1_DA dimers are synclinically tilted layer-by-layer and a schematic unit cell model for this K_M_ phase of AZ_1_DA is illustrated in Fig. 2(d).

### Photochemical isomerization in the SmA phase

**Figure 3:**
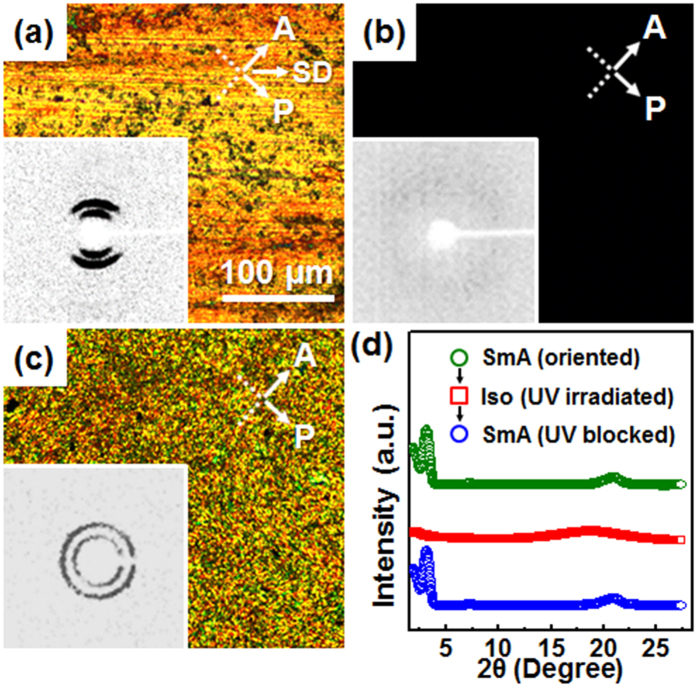
Photochemical isomerization. POM images and their corresponding 2D WAXD patterns (insets, 2θ < 10°) of AZ_1_DA at 100 °C: the macroscopically oriented sample by the mechanical shear (**a**), the isotropic state with the UV light irradiation (**b**) and the ordered sample with multi-domains by turning UV light off (**c**). The corresponding 1D WAXD patterns of AZ_1_DA (**d**).

Since the azobenzene chromophore is applied as the mesogenic group of AZ_1_DA compound, not only the molecular conformations but also the phase structures can be reversibly tuned by the irradiation of appropriate lights. The photochemical behaviors of AZ_1_DA are analyzed by UV-Vis spectroscopy (see Supplementary Fig. S10). The reversible photochemical isomerization of AZ_1_DA can be applied for the light-modulating devices. To realize the optically tunable thin films, the efficient *trans*-to-*cis* and *cis*-to-*trans* photo-isomerization processes of the AZ_1_DA compound should be occurred even in its solid state^32^. As shown in the POM images of Fig. 3(a), it is realized that the SmA phase is transformed to the disordered Iso phase within 30 s under the UV light (365 nm). This photo-induced isothermal phase transition is also monitored by WAXD experiments (inset of Fig. 3). Upon irradiating the UV light, a weak amorphous halo corresponding to the Iso phase is appeared with the simultaneous disappearance of sharp reflection peaks on the meridian (Fig. 3(b)). The layered structure (*L* = 8.61 nm) is totally collapsed by the loss of long-range molecular orders and the dissociation of intermolecular *H*-bondings, which is triggered by the increased amount of *cis*-conformers^33^. When the UV light is blocked, the SmA phase is immediately recovered with the same layer periodicity (Fig. 3(d)). However, both ring diffraction patterns and multi-domain SmA textures clearly display that the original macroscopic orientation of AZ_1_DA is lost (Fig. 3(c)). From this result, the history of molecular orientation can be erased during the photochemical isomerization of AZ_1_DA.

**Figure 4:**
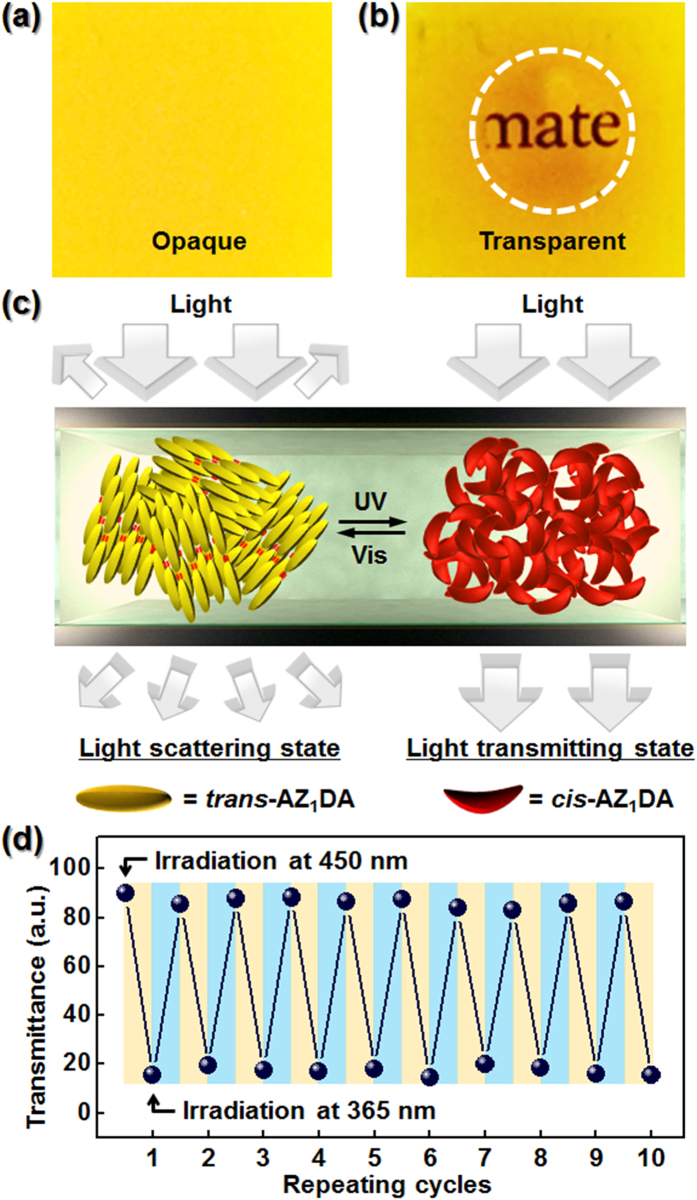
Remote-controllable light shutter. Macroscopic LC cell images of the photo-reversible and photo-patternable thin film from AZ_1_DA upon irradiation of Vis (**a**) and UV (**b**) light. Schematic illustration for photo-induced phase transition of AZ_1_DA (**c**) and its light modulating efficiency (**d**).

While the microphotograph and diffraction patterns in the K_M_ phase are not changed even after the UV light irradiation (see Supplementary Fig. S11). This result clearly indicates that the photo-induced isothermal phase transition is suppressed in the K_M_ phase. Since AZ_1_DA molecules in the highly ordered K_M_ phase are laterally close-packed within the layers, the photo-isomerization from the stable *trans*-conformer (π-π^*^) to the metastable *cis*-conformer (n-π^*^) is fairly limited because of the lack of free volume^34^. The light-triggered phase transition observed in the low-ordered mesophase is owing to the relatively higher mobility of AZ_1_DA than that in the K_M_ phase. Using a versatile photochemical isomerization process in the partially ordered SmA phase, now we successfully demonstrate the light modulating devices from AZ_1_DA molecule. For the fabrication of a remote-controllable light shutter in the LC phase, the AZ_1_DA compound is directly loaded into the optical cell with 10 μm thickness and 10 × 10 mm^2^ lateral dimensions (Fig. 4(a)). The LC cell shows high scatterings at the initial state because of the multi-domain SmA structures. When the LC cell is illuminated by the 365 nm UV light through the round shape photomask, the transparent circular patterns are addressed and the letter in the background, “material” is clearly displayed (Fig. 4(b)). Because the refractive index in the Iso state is independent of the incident angle of light, the light shutter transmits the incident light. While the irradiated UV light is turn off, the transparent state of LC cell immediately returns back to the initial scattering state. The corresponding photochemical isomerization of AZ_1_DA in the SmA ↔ Iso transitions are schematically represented in the Fig. 4(c). The Vis exposed region is transformed from Iso to SmA phase due to the *cis*-to-*trans* isomerization. Additionally, the transmission amplitude is not decayed more than 10 cycles (Fig. 4(d)). Therefore, the optical LC cell of AZ_1_DA shows excellent photo-reversible switching of light across the visible wavelength region by alternating UV and Vis light irradiations without significant hysteresis.

### Topochemical polymerization in the K_M_ Phase

**Figure 5:**
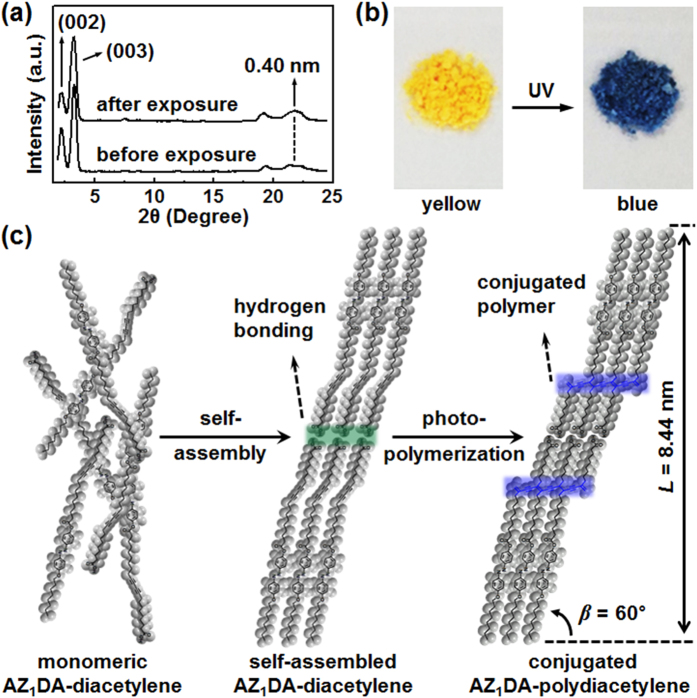
Topochemical polymerization. 1D WAXD patterns of AZ_1_DA-diacetylene and AZ_1_DA-polydiacetylene (**a**) and their photographs of color changes of crystalline AZ_1_DA powder by the 254 nm UV light exposure for 30 min (**b**). Schematic illustrations of molecular packing structures from AZ_1_DA-diacetylene to AZ1DA-polydiacetylene via the supramolecular self-assembly (**c**).

The topochemical polymerization of AZ_1_DA is carried out in the solid state by irradiating UV light (254 nm) at room temperature. It is well known that the photo-polymerization of diacetylene takes place in the appropriate condition that the distances between the adjacent carbons should be below 0.40 nm^35^. As we discussed above in Fig. 2, the self-assembled AZ_1_DA at room temperature shows the highly ordered Cr phase. Therefore, the topochemical polymerization should be achieved in the K_M_ phase of AZ_1_DA. To understand the molecular packing structures of the photo-polymerized AZ_1_DA, the WAXD data are collected before and after the photo-polymerization (Fig. 5(a)). Since the volume creations and the conformational changes would take an enormous amount of energy during the topochemical polymerization, the molecular packing structure of AZ_1_DA-polydiacetylene should be similar to that of the self-assembled AZ_1_DA-diacetylene at 25 °C. As we expected, the diffraction pattern is not changed even after the UV irradiation.

To confirm the topochemical polymerization of AZ_1_DA molecules, UV-Vis spectroscopy measurements are conducted in the bulk state (see Supplementary Fig. S12). The initial AZ_1_DA sample only shows the intensive absorption in the range of 300–500 nm, which can be attributed to the azobenzene chromophore. Upon exposing the 254 nm UV light, the additional absorption band at λ_max_ = 630 nm increases with the irradiation time. The broad absorption band around 500–700 nm is assigned to be the electronic transition of π orbitals from the polydiacetylene backbone^36^. As shown in Fig. 5(b), the color of the AZ_1_DA compound is dramatically changed from yellow to blue right after the topochemical polymerization. This peculiar color change of crystalline AZ_1_DA powder is originated from the formation of conjugated polydiacetylene chains with the carbon-carbon double and triple bonds^37^. Therefore, the absorption spectral change of AZ_1_DA-diacetylene in the K_M_ phase with respect to the 254 nm UV light irradiation fully ensures the formation of AZ_1_DA-polydiacetylene as the consequence of 1,4-addition reaction of the diyne groups of AZ_1_DA compounds. The photo-polymerization of AZ_1_DA is saturated by exposing the UV light for 30 min. Meanwhile, there is no noticeable color and spectral changes in the SmA phase after the 254 nm UV light exposure (see Supplementary Fig. S13). It is obvious that the photo-polymerization of AZ_1_DA in the SmA LC phase is not occurred because the liquid-like order of azobenzene mesogens in the layer disturbs the close packing of diacetylene groups.

The possible molecular packing structure for the AZ_1_DA-polydiacetylene in the K_M_ phase is illustrated in Fig. 5(c). Because of the amphiphilic nature, AZ_1_DA compounds are intrinsically phase-separated to aggregate the supramolecular arrays via the intermolecular *H*-bondings. The densely packed 3D structure in the self-assembled AZ_1_DA-diacetylene is the main condition to provide the facile topochemical polymerization^38^. As noted in Fig. 5(a), the photo-polymerization of diacetylene unit does not have a significant effect on the crystalline structure. The newly developed broad diffraction in the high-angle region should be related with the created carbon-to-carbon bonds within the AZ_1_DA-polydiacetylene backbones. The low-angle reflection clearly indicates that the layer structures still exist due to the head-to-head dimerization. Since the conjugated AZ_1_DA-polydiacetylene restricts the conformational mobility of backbones, the highly ordered layer structure with the monoclinic lattice is preserved even after the topochemical polymerization process.

**Figure 6:**
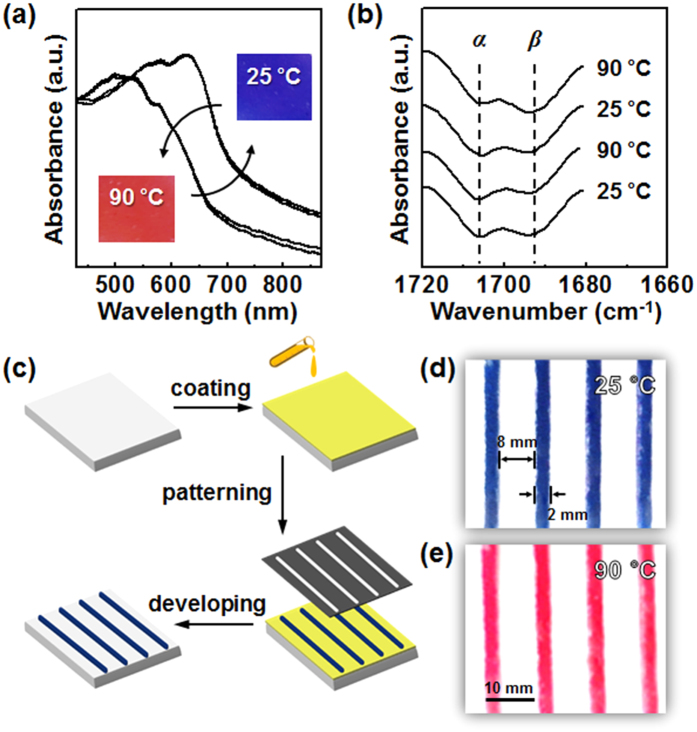
Thermo-responsive color switch. Changes of UV-Vis (**a**) and FT IR (**b**) spectra for the AZ_1_DA-polydiacetylene film upon thermal cycles. Fabrication procedures of the photo-patternable thin films (**c**). Photographs of colorimetric sensors at 25 °C (**d**) and 90 °C (**e**), respectively.

Interestingly, the AZ_1_DA-polydiacetylene shows the reversible thermochromic behaviors. It is here worth mentioning the fact that the color transition of polydiacetylene is caused by varying the effective conjugation length resulting from the distortions of backbone conformations^39^. The color transition of AZ_1_DA-polydiacetylene are recorded by a digital camera upon heating and subsequent cooling processes (inset of Fig. 6(a)). The initial blue color of AZ_1_DA-polydiacetylene film turns to red upon heating. For a better insight into the thermochromism, the temperature-dependent absorption change is studied with UV-Vis spectroscopy (Fig. 6(a)). As heating from 25 °C to 90 °C, the absorption peak at 540 nm is increased by concomitantly decreasing the absorption peak at 630 nm, corresponding to the blue to red shift. Note that the light of particular wavelength is absorbed and the rest of light passes through to reach the eye. When the heat is applied to the AZ_1_DA-polydiacetylene, the accumulated stress of side-chain during the topochemical polymerization is released by partial disorder of alkyl chains in the statistically distributed *trans* and *gauche* conformations. Eventually, twisting the π orbitals to the non-planar states reduces the effective conjugation length which results in the red shift^40^.

When the film is subsequently cooled back to 25 °C, the original spectrum is completely recovered corresponding to the blue appearance of the film. It is well established that the *H*-bonding maintained throughout the thermal fluctuation is an essential requirement for the reversible thermochromism of AZ_1_DA-polydiacetylene supramolecules^41^. As shown in Fig. 6(b), the C = O stretching bands are observed in the ester group at the *α* position (1705 cm^−1^) and the carboxyl group at the *β* position (1695 cm^−1^), respectively. The *H*-bonded carbonyl stretching of AZ_1_DA-polydiacetylene retains during the heating (90 °C) and cooling (25 °C) cycles as indicated by the absence of an intensity change as well as by the positional change at the *β* peak. This finding demonstrates that *H*-bond is not altered during the thermal stimulus at the given condition. From the perspective view of the molecular packing of AZ_1_DA-polydiacetylene, the confined geometry in the highly ordered layer structure with the monoclinic lattice is helpful to construct the bilayered structure against the thermal stimuli. Therefore, the original state of effective conjugation length can be restored when the thermal stress is eliminated.

We also made an effort to fabricate the patterned color film for the thermochromic sensor. For this purpose, a photo-lithographic method is employed. First, the AZ_1_DA films are prepared by the drop-casting of the AZ_1_DA in chloroform solution and the subsequent thermal annealing at 80 °C. Second, the prepared thin film is irradiated with the 254 nm UV light for 30 min through a strip patterned photomask. Note that the once photo-polymerized diacetylenes are not soluble in common organic solvents owing to its chemical cross-linking^42^. The unpolymerized part which is selected by the photomask can be effectively removed by the developing process in good solvents. The overall fabrication procedures are schematically illustrated in Fig. 6(c). As shown in Fig. 6(d), the intensive striped blue patterns with a good fidelity have been observed in macroscopic views at 25 °C. The UV exposed regions are blue, confirming the successful photo-polymerization. As anticipated, the corresponding red patterned film is obtained by the subsequent heating process to 90 °C (Fig. 6(e)). The thermochromic reversibility of this film retains far beyond 10 times heating and cooling cycles without any apparent reductions.

## Conclusion

Understanding and constructing the strong relationships between chemical structures, molecular packing structures and physical properties of the programmed soft materials is the core research of smart and multifunctional materi als. For the fabrication of well-defined hierarchical superstructures on the different length scales via the self-assembly processes, a dual photo-functionalized amphiphile (abbreviated as AZ_1_DA) was newly designed and synthesized by chemically connecting the photo-isomerizable azobenzene and the photo-polymerizable diacetylene. Based on the careful investigation with thermal, microscopic, spectroscopic, scattering and simulation techniques, it was realized that the AZ_1_DA molecules basically formed the layer structure with 8.61 nm periodicity in the smectic A (SmA) LC phase at higher temperatures. At lower temperatures, the highly ordered crystalline phase was found with the monoclinic (K_M_) lattice parameters of *a* = 0.61 nm, *b* = 0.90 nm, *c* = 9.74 nm and *α* = *γ* = 90° and *β* = 60°. Light-induced phase transformations of AZ_1_DA in SmA and K_M_ phases provided us a lot of opportunities for the fabrication of remote-controllable optical devices. The light-modulating device was fabricated by applying the photo-reversible *trans*-*cis* isomerization of azobenzene group in the SmA phase, while the thermo-reversible color switching was demonstrated via the photo-polymerization of diacetylene groups in the highly ordered K_M_ phase.

## Methods

### Synthesis

Solution of 4-(4′-octyloxy)hydroxyazobenzene (1.0 eq.), 10,12-docosadiynedioic acid (2.0 eq.), dicyclohexylcarbodiimide (DCC, 2.0 eq.) and 4-(dimethylamino) pyridine (DMAP, 0.2 eq.) in anhydrous tetrahydrofuran was stirred at room temperature for 72 h. After reaction, solvent was distilled off and remaining residue was re-dissolved in chloroform and washed with water three times. The crude product was purified by column chromatography on silica gel using ethyl acetate:methylene chloride = 1:3. Resulting product was pale yellow solid (yield: 72%). ^1^H NMR (400 MHz, CDCl_3_): δ 0.89 (t, 3H), 1.23–1.51 (m, 30H), 1.62 (m, 2H), 1.85 (m, 4H), 2.24 (q, 4H), 2.34 (t, 2H), 2.60 (t, 2H), 4.03 (t, 2H), 7.00 (d, 2H), 7.20 (d, 2H), 7.89 (m, 4H); ^13^C NMR (400 MHz, CDCl_3_): δ 172.0, 161.7, 152.2, 150.2, 146.7, 124.7, 123.6, 122.1, 114.7, 77.4, 68.3, 65.3, 34.4, 33.7, 31.8, 28.7, 28.2, 26.0, 24.8, 24.6, 22.6, 19.1, 14.1 ppm; MALDI-ToF MS (m/z): [M]^+^ calcd. for 670.92; [M+Na]^+^ found for 693.91; elemental analysis (calcd., found for C_42_H_58_N_2_O_5_): C (75.19, 75.08), H (8.71, 8.64), N (4.18, 4.18).

### Characterization

The AZ_1_DA compound was always kept in a vacuum before carrying out the analysis. The ^1^H and ^13^C NMR spectra was recorded on a spectrometer (JNMEX400) in CDCl_3_. The MALDI-ToF (Voyager DE) and elemental analysis (Vario EL) was conducted to identify the chemical structure and purity. For the DSC (PerkinElmer PYRIS) experiments, the sample weight was about 5.0 mg and the pan weights were kept constant with a precision of ±0.001 mg. In order to identify the phase structures, AZ_1_DA films with a thickness = 0.7 mm were prepared by melting the compounds in an aluminum cell. The 1D WAXD and 1D SAXS experiments were conducted by utilizing the Cu Kα radiation generator with a diffractometer (Rigaku). The oriented samples were made by mechanically shearing the sample. The 2D WAXD patterns were obtained by using an imaging system with X-ray generator (Rigaku). The change of optical texture was observed by using POM (Nikon ECLIPSE) coupled with a heating stage (Linkam LTS). The Cerius^2^ simulation software (Accelrys version 4.6) was used to calculate the minimal-energy geometry. The UV-Vis absorption spectra were obtained with a spectrophotometer (SCINCO S-3100). The photographic images were taken using a digital camera (Nikon D5300).

## Additional Information

**How to cite this article**: Kim, D.-Y. *et al*. Photochemical Isomerization and Topochemical Polymerization of the Programmed Asymmetric Amphiphiles. *Sci. Rep.* **6**, 28659; doi: 10.1038/srep28659 (2016).

## Supplementary Material

Supplementary Information
