# Supplementary material for: Photochemical Isomerization and Topochemical Polymerization of the Programmed Asymmetric Amphiphiles
Source: Sci Rep. 2016 Jun 24;6:28659. doi: 10.1038/srep28659 (PMC4919645; doi:10.1038/srep28659)
Supplement: Supplementary Information [file srep28659-s1.pdf]

Supplementary Information

Photochemical Isomerization and Topochemical Polymerization of the Programmed Asymmetric Amphiphiles

Dae-Yoon Kim, Sang-A Lee, Daseal Jung & Kwang-Un Jeong*

Polymer Materials Fusion Research Center & Department of Polymer-Nano Science and Technology, Chonbuk National University, Jeonju, Jeonbuk, 561-756, Korea.

Correspondence and requests for materials should be addressed to K.U.J. (E-mail: kujeong@jbnu.ac.kr)

1. Materials

4-nitrophenol (Aldrich), 1-bromooctane (Aldrich), hydrazine monohydrate (N_2H_4 H_2O, Sigma-Aldrich), Raney-Nickel catalyst (Al/Ni, Aldrich), phenol (PhOH, Aldrich), 10,12-docosadiynedioic acid (Alfa Aesar), *N,N*-dicyclohexylcabodiimide (DCC, Aldrich) and 4-(dimethylamino)pyridine (DMAP, Aldrich) was used as received.

2. Synthesis

1-nitro-4-(octyloxy)benzene (1). A solution of 4-nitrophenol (20.7 mmol) and 1-bromooctane (20.7 mmol) and K_2CO_3 (49.7 mmol) in 50 mL of dried acetone was refluxed for 6 h. After reaction, the solvent was removed in vacuum and the residue was dissolved in chloroform and washed with distilled water several times. The organic layer was dried over $MgSO_4$. It was purified by column chromatography with silica gel using chloroform:hexanes = 1:2. Resulting product was viscous and yellowish liquid (yield: 81%). ^1H NMR (400 MHz,

$CDCl_3$, TMS): δ = 0.89 (t, 3H), 1.23-1.51 (m, 10H), 1.80 (m, 2H), 4.02 (t, 2H), 6.93 (d, 2H), 8.18 (d, 2H) ppm.

4-(octyloxy)benzenamine (2). N_2H_4 H_2O (20.3 mmol) was added dropwise to the solution of **1** (11.2 mmol) in 40 mL of absolute ethanol. After the solution was heated to 45 °C, catalytic amount of the activated Al/Ni was added until no further reaction was observed. The resulting mixture was filtered and the ethanol was removed under reduced pressure. The crude was dissolved in diethylether, washed with water, and dried with $MgSO_4$. The brownish product was obtained by distillation (yield: 94%). ^1H NMR (400 MHz, $CDCl_3$, TMS): δ = 0.89 (t, 3H), 1.23-1.51 (m, 10H), 1.80 (m, 2H), 3-3.7 (s, 2H), 4.02 (t, 2H), 6.63 (d, 2H), 6.75 (d, 2H) ppm.

4-(4'-octyloxy)hydroxyazobenzene (3). Below 5 °C, a 2.5 M $NaNO_2$ solution was added to a heterogeneous mixture of **2** (32.6 mmol) in 5.0 M HCl. The mixture at 5 °C was carefully added into the solution of PhOH (32.9 mmol) in 2.0 M NaOH. The precipitated reddish powder was filtered and pure product was obtained by the recrystallization in hexane (yield: 72%). ^1H NMR (400 MHz, $CDCl_3$, TMS): δ = 0.89 (t, 3H), 1.23-1.51 (m, 10H), 1.79 (m, 2H), 4.03 (t, 2H), 6.92 (d, 2H), 6.98 (d, 2H), 7.85 (q, 4H) ppm.

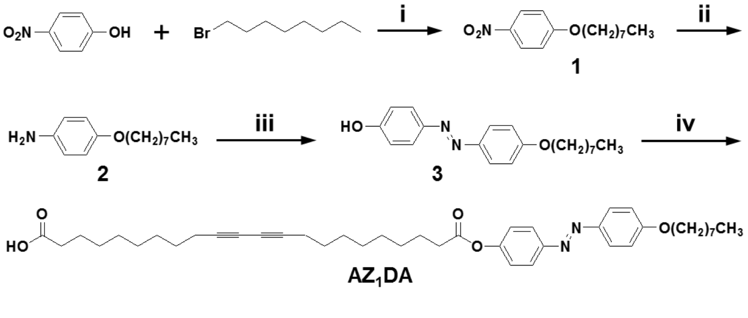

Figure S1. Synthetic procedures of AZ_1DA. Reagents and conditions: (i) K_2CO_3, acetone, reflux, 6 h; (ii) N_2H_4 H_2O, Al/Ni, EtOH, reflux, 1 h; (iii) HCl, $NaNO_2$, NaOH, PhOH, 5 °C, 1 h; (iv) 10,12-docosadiynedioic acid, DCC, DMAP, THF, 25 °C, 24 h.

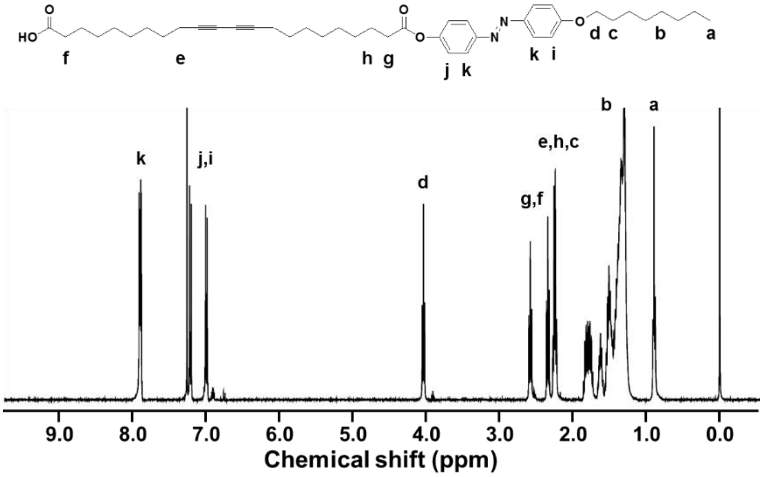

Figure S2. ^{1}H NMR spectrum of AZ_1DA.

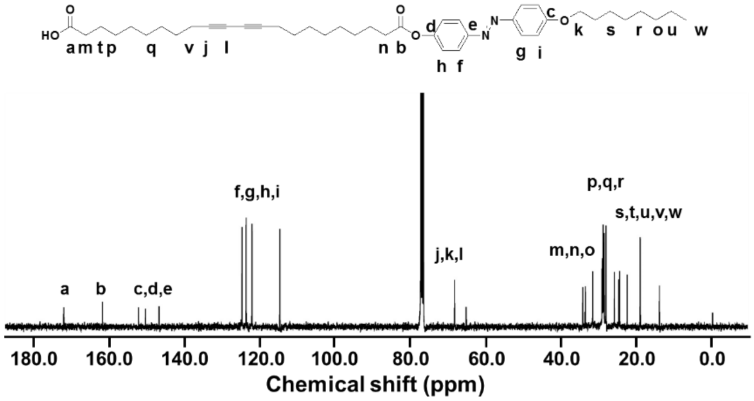

Figure S3. ^{13}C NMR spectrum of AZ_1DA.

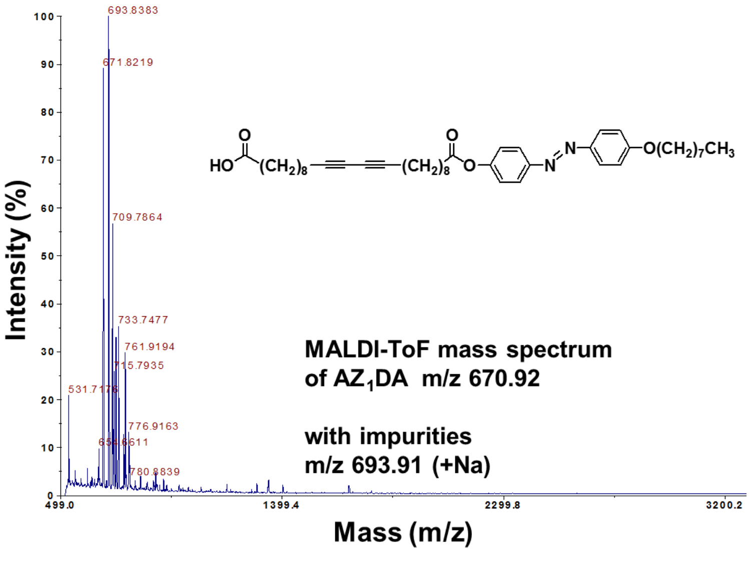

Figure S4. MALDI-ToF MASS spectrum of AZ_1DA.

	Experimental Content (%)	Calculated Content (%)
C	75.08<br>75.12<br>75.54	75.19
H	8.647<br>8.639<br>8.656	8.71
N	4.181<br>4.115<br>4.161	4.18

Figure S5. Elemental analysis of AZ_1DA.

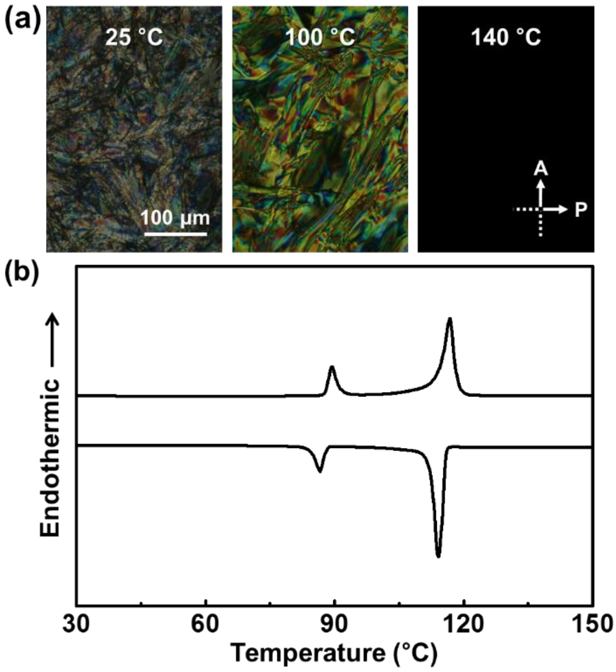

Figure S6. POM images at different temperature (a) and DSC thermograms during cooling and heating of AZ_1DA compound.

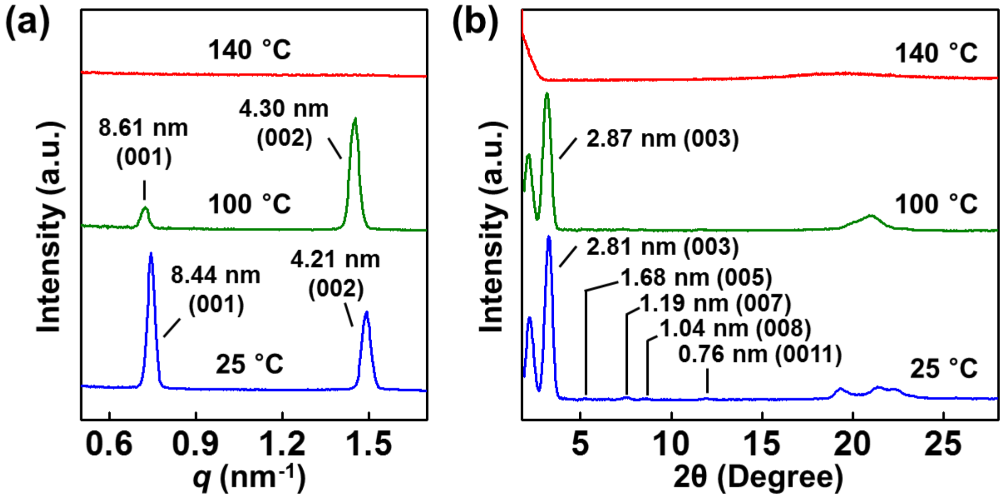

Figure S7. 1D SAXS (a) and 1D WAXD (b) of AZ_1DA compound at different temperatures.

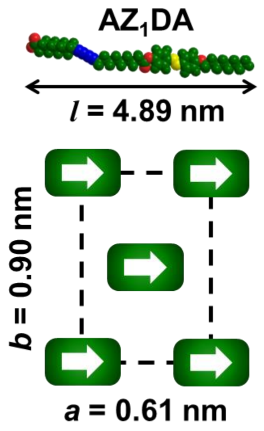

Figure S8. Energy minimized molecular geometry and schematic illustration of the molecular arrangement in the K_M phase of AZ_1DA.

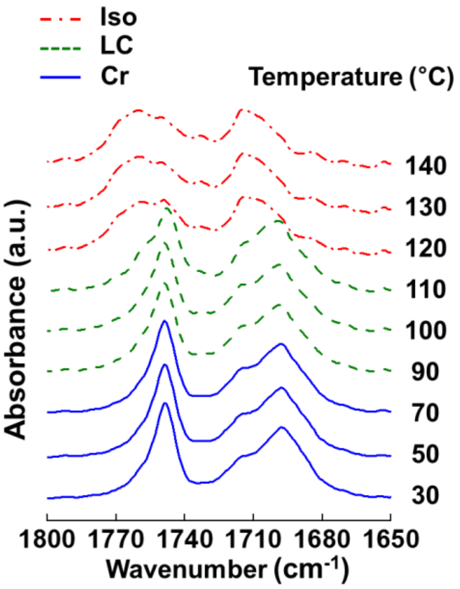

Figure S9. FT IR spectra of AZ_1DA compound between 1650 and 1800 cm^{-1} at different temperatures.

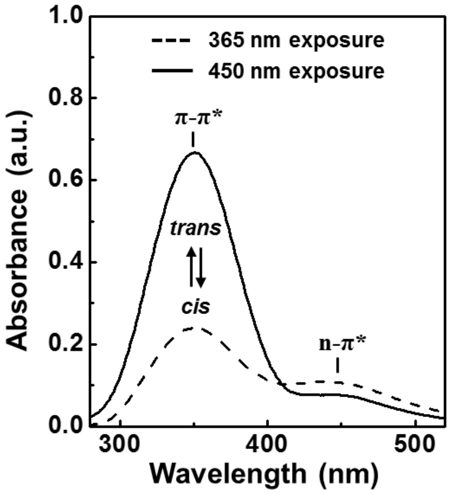

Figure S10. UV-Vis absorption spectra of the AZ_1DA compound obtained upon irradiating 365 nm and 450 nm lights.

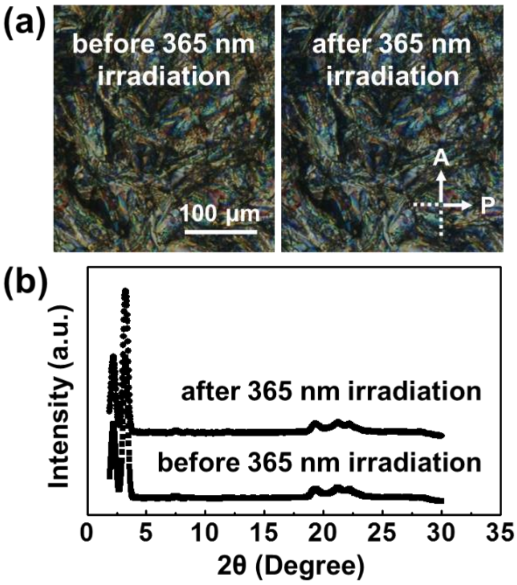

Figure S11. POM images (a) and 1D WAXD patterns (b) of the AZ_1DA compound upon the 365 nm UV irradiation at 25 °C.

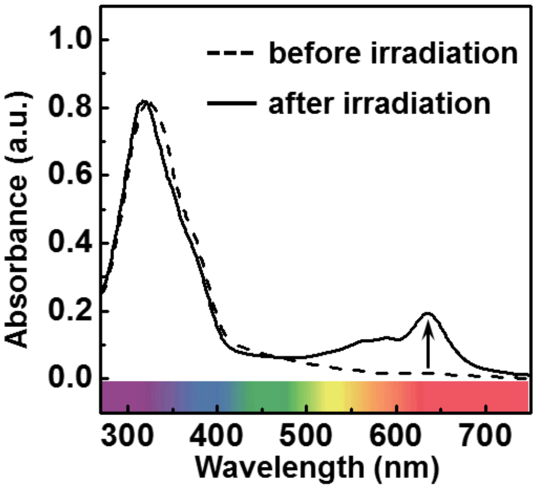

Figure S12. UV-Vis absorption spectral changes of the AZ_1DA compound via the topochemical polymerization by the 254 nm UV light exposure for 30 min.

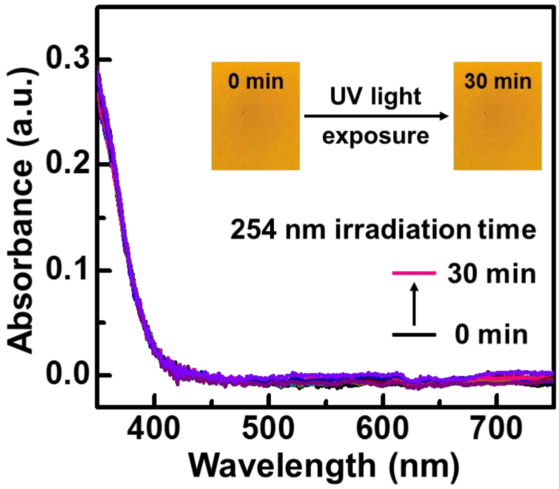

Figure S13. Absorption spectra and photographic images of the AZ_1DA compound upon the 254 nm UV irradiation at 100 °C.
